# Nicotine Inhibits the Cytotoxicity and Genotoxicity of NNK Mediated by CYP2A13 in BEAS-2B Cells

**DOI:** 10.3390/molecules27154851

**Published:** 2022-07-29

**Authors:** Yulin Sun, Hongjuan Wang, Huan Chen, Sen Zhang, Jun Li, Jingni Zhang, Jianlu Tian, Youyu Zhang, Hongwei Hou, Qingyuan Hu

**Affiliations:** 1 China National Tobacco Quality Supervision & Test Center, Zhengzhou 450001, China; sunyl@tbescience.com (Y.S.); redbri2013@163.com (H.W.); hunny_ch@163.com (H.C.); lijun@tbescience.com (J.L.); zhangjn@tbescience.com (J.Z.); tianjl@tbescience.com (J.T.); 2 Key Laboratory of Tobacco Biological Effects, Zhengzhou 450001, China; 3 Key Laboratory of Chemical Biology and Traditional Chinese Medicine Research (Ministry of Education of China), College of Chemistry and Chemical Engineering, Hunan Normal University, Changsha 410081, China; 4 Shaanxi Key Laboratory of Degradable Biomedical Materials, School of Chemical Engineering, Northwest University, Xi’an 710069, China; liqvanzs@163.com

**Keywords:** NNK, nicotine, cytotoxicity, genotoxicity, cytochrome P450 2A13

## Abstract

Both tobacco-specific carcinogen 4-(methylnitrosamino)-1-(3-pyridyl)-1-butanone (NNK) and nicotine can be metabolized by cytochrome P450 2A13 (CYP2A13). Previous studies have shown that nicotine has a potential inhibitory effect on the toxicity of NNK. However, due to the lack of CYP2A13 activity in conventional lung cell lines, there had been no systematic in vitro investigation for the key target organ, the lung. Here, BEAS-2B cells stably expressing CYP2A13 (B-2A13 cells) were constructed to investigate the effects of nicotine on the cytotoxicity and genotoxicity of NNK. The results showed more sensitivity for NNK-induced cytotoxicity in B-2A13 cells than in BEAS-2B and B-vector cells. NNK significantly induced DNA damage, cell cycle arrest, and chromosomal damage in B-2A13 cells, but had no significant effect on BEAS-2B cells and the vector control cells. The combination of different concentration gradient of nicotine without cytotoxic effects and a single concentration of NNK reduced or even counteracted the cytotoxicity and multi-dimensional genotoxicity in a dose-dependent manner. In conclusion, CYP2A13 caused the cytotoxicity and genotoxicity of NNK in BEAS-2B cells, and the addition of nicotine could inhibit the toxicity of NNK.

## 1. Introduction

4-(Methylnitrosamino)-1-(3-pyridyl)-1-butanone (NNK), the strongest carcinogen of tobacco-specific N-nitrosamines (TSNAs) in cigarette smoke, is listed as a Group 1 human carcinogen by the International Agency for Research of Cancer. NNK is also a key component of monitored tobacco carcinogens and is thought to play an important role in smoking-related cancers in humans [1,2,3,4]. NNK must be metabolically activated to exert its carcinogenic and toxicological properties [2,5]. It is difficult to study the toxic effects of NNK due to the complexity of NNK metabolism, including the enzyme system and activation process of the catalysis [1]. NNK leads to the α-hydroxylation of methyl or methylene carbon via bioactivation of cytochrome P450 enzymes (CYP), and CYP2As play pivotal roles in the metabolic activation of NNK in the human pulmonary system, such as CYP2A6, CYP2A7, and CYP2A13. Among these, CYP2A7 is defective in incorporating heme molecules and therefore is an inactive enzyme in vitro. The tissue distribution of these enzymes revealed that CYP2A6 is mainly expressed in the liver, and a minimally in the lungs, and CYP2A13 is predominantly expressed in the respiratory tract, including lung, trachea, and nasal mucosa [6,7]. The metabolism of NNK form unstable intermediates that are then broken down into electrophilic methyl-, pyridine-, oxa-nbutyl-, and pyridylhydroxybutyl-diazonium ions. These ions may attack DNA molecules, and lead to the formation of methyl and butylpyridoxine DNA adducts, other DNA damage and chromosome damage, which could be involved in the toxicological properties of NNK [7,8].

Of all the human CYP enzymes detected thus far, CYP2A13 is the most active and efficient enzyme for NNK metabolic activation in vitro and in vivo [2,9]. CYP2A13 is also a catalyst for nicotine, which is the main alkaloid in tobacco and the primary addictive substance in cigarette smoke, maintaining the long-term use of tobacco products [10,11]. Previous studies showed that irreversible inactivation of CYP2A13 enzyme activity occurs in a time and dose-dependent manner after nicotine metabolism [12]. This affects the metabolic activation of NNK, and then affects the toxic effect of NNK, so it is of great significance to explore the effect of nicotine on the toxicity of NNK. Nicotine has been reported to prevent NNK-induced DNA damage in human hepatic-derived cells, possibly by inhibiting CYP enzymes [13]. However, that study only used a single genotoxicity index to investigate, and did not conclusively demonstrate that nicotine inhibited NNK-induced DNA damage mediated by CYP enzyme. NNK is a more potent lung carcinogen than that of the liver [14], and the main target organ for cancer caused by NNK is the lungs [15]. Therefore, it is necessary to study the effect of nicotine on NNK-induced toxicity in the lung, especially genotoxicity. 

Typically, the ideal system to study lung carcinogenicity would be airway cells with a metabolic capacity [13]. Immortalized human bronchial epithelial (BEAS-2B) cells can be used as a substitute in toxicological and pharmacological studies due to their relatively high homology to human lung tissues and primary cells in gene expression patterns [15], but studies have demonstrated low activity and low expression of CYP2A13 in BEAS-2B cells [16]. Moreover, the CYP enzymes required for NNK metabolism are lacking in conventional test lung cell lines [16,17]. Thus, this study established genetic engineered BEAS-2B cells that could stably express CYP2A13 (B-2A13) using the lentiviral system, and BEAS-2B cells and the vector control cells were used as the control. Based on these cell lines, this study explored the role of CYP2A13 in cytotoxicity and genotoxicity of NNK and the effect of nicotine on the toxic effects induced by NNK in lung cells.

## 2. Results and Discussion

Tobacco is a potent multisite carcinogen with a substantial worldwide impact [18]. Both nicotine and NNK are important components in cigarette smoke that contribute to cancer development [19,20], and nicotine has been reported to inhibit the metabolism of NNK by inhibiting CYP2A13 [21,22], so it is of great significance to investigate the effect of CYP2A13-mediated nicotine on the cytotoxicity and genotoxicity of NNK on lung cells. Therefore, based on the constructed B-2A13 cells, the toxic effects of NNK alone and NNK combined with nicotine were studied in this study.

### 2.1. Identification of BEAS-2B Cells That Stably Express CYP2A13

**Figure 1:**
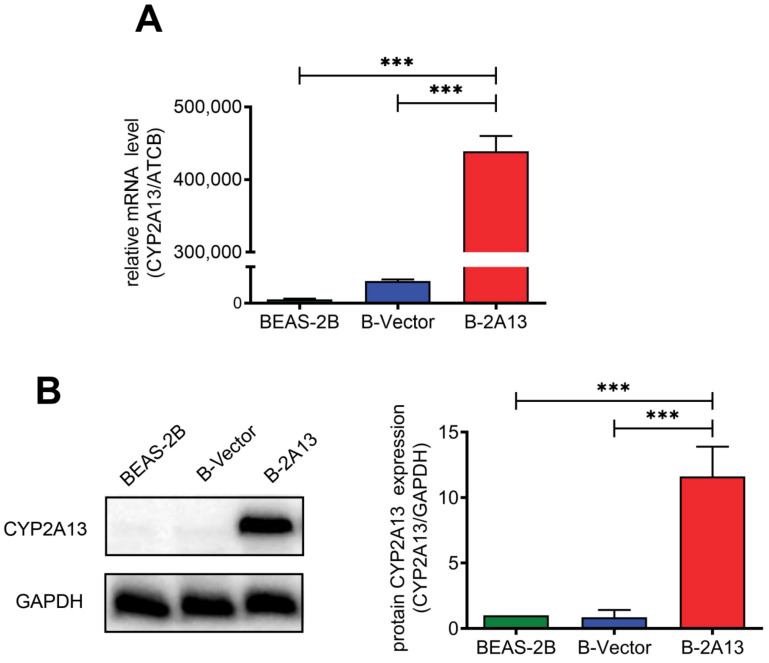
Identification of BEAS-2B cells that stably express the cytochrome P450 2A13 (CYP2A13). (**A**) mRNA expression of CYP2A13 in cells determined with quantitative Real-Time PCR (qRT-PCR). (**B**) Characterization of CYP2A13 protein expression in cell lysates. The protein levels of CYP2A13 were detected by Western blotting. Beta-actin gene (ATCB) gene served as a reference gene in the qRT-PCR experiment. Glyceraldehyde-3-phosphate dehydrogenase (GAPDH) served as a reference protein in the WB experiment. ***: *p* < 0.001.

BEAS-2B cells stably expressing CYP2A13 (B-2A13) or vector (B-Vector) were constructed using the lentiviral system. The expression abundance of mRNA of the CYP2A13 gene in BEAS-2B cells, B-Vector cells, and B-2A13 cells was determined by quantitative Real-Time PCR (qRT-PCR), and the results showed that the expression abundance of the CYP2A13 in B-2A13 cells was significantly higher than that in B-Vector and BEAS-2B cells (*p* < 0.001) (Figure 1A). For example, the expression abundance of the CYP2A13 gene in B-2A13 cells was 438,836.7 times higher than that in BEAS-2B cells (Figure 1A). Western blotting showed that the single CYP2A13 protein band was detected in B-2A13 cells, while there were no obvious bands observed in the B-Vector and BEAS-2B cells (Figure 1B). This indicates that CYP2A13 was successfully expressed in BEAS-2B cells, which was consistent with the results obtained by qRT-PCR. It indicated that BEAS-2B cells stably expressing CYP2A13 were successfully established. 

### 2.2. Effects of Nicotine on NNK-Induced Cytotoxicity in B-2A13 Cells

**Figure 2:**
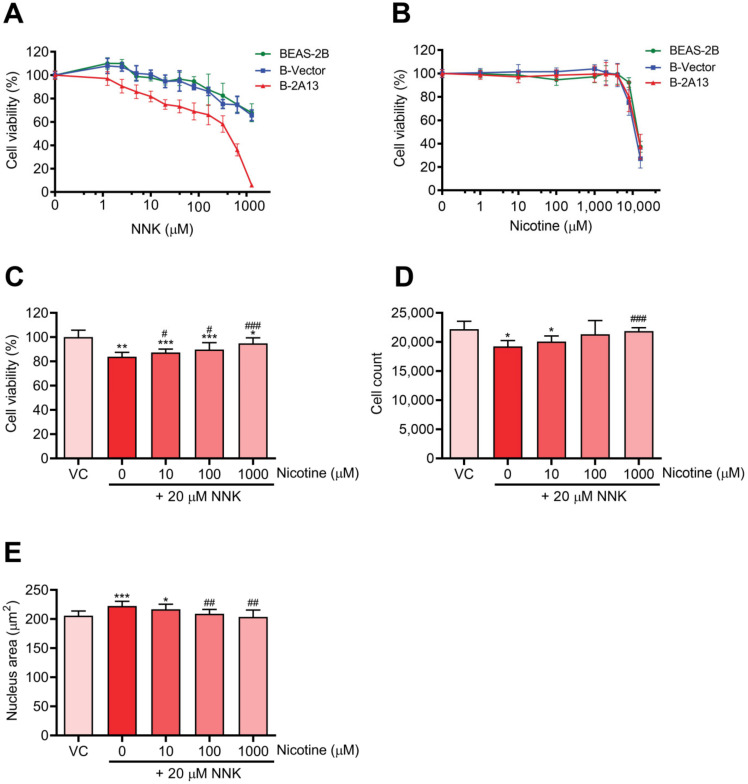
Nicotine inhibited 4-(methylnitrosamino)-1-(3-pyridyl)-1-butanone (NNK)-induced cytotoxicity in B-2A13 cells. Cells were treated with NNK and/or nicotine for 24 h. Cell viability of BEAS-2B, B-Vector, and B-2A13 cells after treatment with NNK (**A**) and nicotine (**B**). B-2A13 cells had no significant effects on the cell viability (*p* > 0.05). Cell viability (**C**), cell count (**D**), and nucleus area (**E**) of B-2A13 cells after treatment with a combination of NNK and nicotine. Vehicle control (VC) was cells treated with dissolved in dimethyl sulfoxide (DMSO) (0.004%). * Shows comparison with VC group, * *p* < 0.05, ** *p* < 0.01, and *** *p* < 0.001; # Shows comparison with 20 μM of NNK group, # *p* < 0.05, ## *p* < 0.01, and ### *p* < 0.001.

To obtain suitable dosing concentration ranges for nicotine and NNK, cytotoxicity was first evaluated using the Cell Counting Kit-8 (CCK-8) assay. NNK induced cytotoxicity in B-2A13 cells in a dose-dependent manner, and B-2A13 cells were more sensitive to NNK than B-Vector cells and BEAS-2B cells (Figure 2A). After 24 h of treatment, the IC_20_ value of NNK was 24.52 μM, 265.04 μM, and 376.14 μM for B-2A13, B-Vector, and BEAS-2B cells, respectively. These results showed that CYP2A13 played an indispensable mediating role in NNK-induced cytotoxicity.

The viability of cells exposed to nicotine is shown in Figure 2B. Effects of nicotine on the viability of BEAS-2B, B-Vector, and B-2A13 cells were similar. There was no significant difference in the cell viability after 24 h of treatment with nicotine (10–1000 μM) compared to the control group (*p* > 0.05) in B-2A13 cells. Accordingly, 10–1000 μM of nicotine was selected for further experiments for the co-exposure of nicotine and NNK.

Nicotine reduced NNK-induced inhibition of cell viability in B-2A13 cells in a dose-dependent manner (Figure 2C). In the High content screening (HCS) approach, after 24 h of treatment with 20 μM of NNK in B-2A13 cells, the number of cells significantly decreased (Figure 2D, *p* < 0.05), and the area of the nucleus significantly increased (Figure 2E, *p* < 0.001). However, these adverse changes were mitigated in all the groups co-incubated with nicotine. 

To avoid false positive genotoxic signals due to cell apoptosis or necrosis, experimental compounds or mixtures are generally considered to be genotoxic when the cell viability of the experimental compound or mixture is greater than 80%, and the induction of H2AX phosphorylation is at least 20% higher than that of the control [23,24]. Therefore, this study used 20 μM of NNK for the combined exposure with nicotine, and the cytotoxicity of this concentration was not higher than 20% in B-2A13 cells. To investigate the effects of nicotine with different concentration gradients on the cytotoxicity and genotoxicity of NNK, three noncytotoxic concentrations of nicotine (10, 100, and 1000 μM) were selected.

### 2.3. Effects of Nicotine on NNK-Induced Genotoxicity and Cell Cycle Arrest in B-2A13 Cells

**Figure 3:**
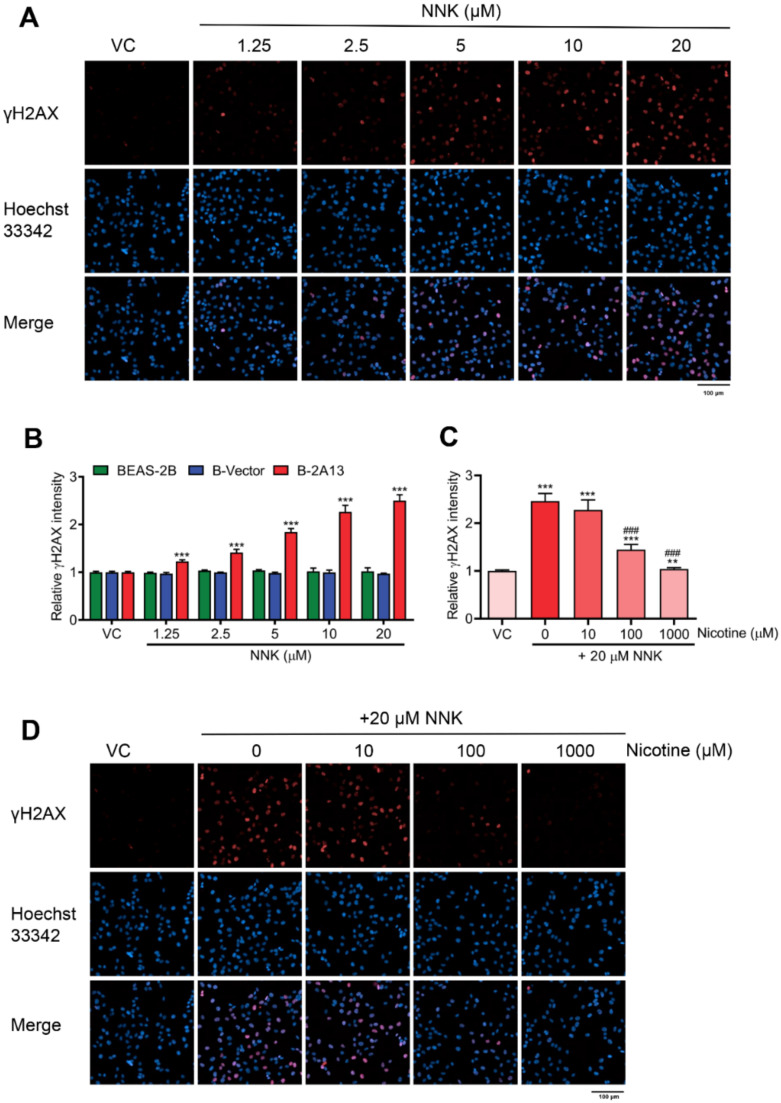
Nicotine inhibited NNK-induced DNA damage in B-2A13 cells. DNA damage was determined using γH2AX assay with high content screening. Cells were treated with NNK alone or a combination of NNK and nicotine for 24 h. (**A**) Immunostaining and imaging analysis of γH2AX in B-2A13 cells treated with NNK. (**B**) Relative γH2AX intensity in BEAS-2B, B-Vector, and B-2A13 cells treated with NNK. (**C**) Relative γH2AX intensity in B-2A13 cells treated with a combination of NNK and nicotine. (**D**) Immunostaining and imaging analysis of γH2AX in B-2A13 cells treated with a combination of NNK and nicotine. Vehicle control (VC) was cells treated with DMSO (0.004%). * Shows comparison with VC group, ** *p* < 0.01, and *** *p* < 0.001; # Shows comparison with 20 μM of NNK group, ### *p* < 0.001.

γH2AX is widely recognized as a sensitive biomarker of DNA double-strand breaks (DSBs) [25]. DNA damage was determined using γH2AX assay. After 24 h of treatment with 1.25–20 μM of NNK, the fluorescence intensity of γH2AX dose-dependently increased in B-2A13 cells and 1.25 μM of NNK significantly induced γH2AX in B-2A13 cells (*p* < 0.001) compared with the vehicle control, while BEAS-2B cells and B-vector cells had no significant effects on NNK-induced γH2AX compared with the vehicle control (Figure 3A,B). These results demonstrated that the involvement of CYP2A13 promoted NNK-induced DNA damage. About 20 μM of NNK increased the fluorescence intensity of γH2AX in cell nuclei of B-2A13 cells, while the groups incubated with nicotine dose-dependently down-regulated the fluorescence intensity of γH2AX in B-2A13 cells (Figure 3C,D). Therefore, nicotine inhibited NNK-induced DNA damage in B-2A13 cells in a dose-dependent manner.

**Figure 4:**
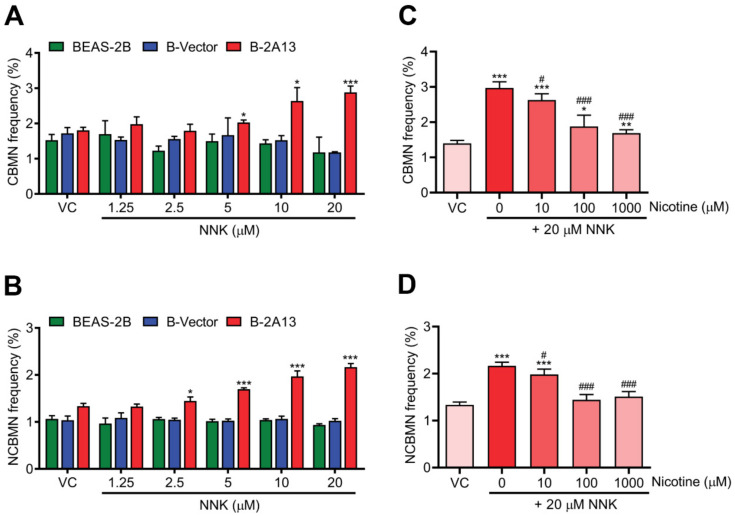
Nicotine inhibited NNK-induced chromosome damage in B-2A13 cells. Chromosome damage was detected using the cytoplasmic block micronucleus (CBMN) and non cytoplasmic block micronucleus (NCBMN) assays with high content screening. Cells were treated with NNK alone or a combination of NNK and nicotine for 24 h. CBMN frequency (**A**) and NCBMN frequency (**B**) in BEAS-2B, B-Vector, and B-2A13 cells treated with NNK. CBMN frequency (**C**) and NCBMN frequency (**D**) in B-2A13 cells treated with a combination of NNK and nicotine. Vehicle control (VC) was cells treated with DMSO (0.004%). * Shows comparison with VC group, * *p* < 0.05, ** *p* < 0.01, and *** *p* < 0.001; # Shows comparison with 20 μM of NNK group, # *p* < 0.05, and ### *p* < 0.001.

The cytoplasmic block micronucleus (CBMN) and non cytoplasmic block micronucleus (NCBMN) assay were used to evaluate chromosome damage. Compared with the vehicle control, CBMN frequency and NCBMN frequency of B-2A13 cells increased in a dose-dependent fashion. About 5 μM NNK significantly increased CBMN frequency (*p* < 0.05) (Figure 4A), and 2.5 μM NNK significantly increased NCBMN frequency in B-2A13 cells (*p* < 0.05) (Figure 4B), whereas no significant changes in CBMN frequency and NCBMN frequency were found in either BEAS-2B or B-Vector cells (*p* > 0.05) (Figure 4A,B). These results demonstrated that chromosomal damage induced by NNK needed mediation by CYP2A13. As expected, nicotine could reduce the CBMN frequency and the NCBMN frequency, and as the concentration increased, the CBMN frequency and the NCBMN frequency returned to the basal level (Figure 4C,D). Thus, nicotine inhibited the CYP2A13-mediated genotoxic effects (DNA damage and chromosome damage) of NNK.

**Figure 5:**
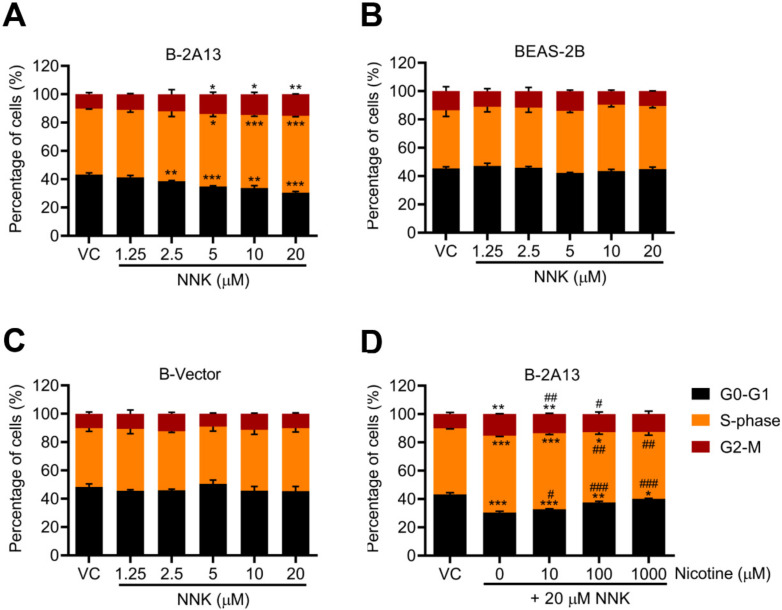
Nicotine inhibited NNK-induced cell cycle arrest in B-2A13 cells. Cells were treated with NNK alone or a combination of NNK and nicotine for 24 h. Cell cycle in B-2A13 cells (**A**), BEAS-2B cells (**B**), and B-Vector cells (**C**) treated with NNK, and cell cycle in B-2A13 cells treated with a combination of NNK and nicotine (**D**). Vehicle control (VC) was cells treated with DMSO (0.004%). * Shows comparison with VC group, * *p* < 0.05, ** *p* < 0.01, and *** *p* < 0.001; # Shows comparison with 20 μM of NNK group, # *p* < 0.05, ## *p* < 0.01, and ### *p* < 0.001.

The percentage of cells in the S-phase and G2-phase increased in a dose-dependent manner after 24 h of NNK treatment in B-2A13 cells (Figure 5A). However, no significant effect of NNK was found on the cell cycle arrest in BEAS-2B cells and B-vector cells (*p* > 0.05) (Figure 5B,C). In concordance with the above results, nicotine restrained NNK-induced cell cycle arrest in B-2A13 cells in a concentration-dependent manner (Figure 5D).

A systemic toxicity assessment of NNK based on normal lung cells stably expressing CYP2A13 had not been previously reported. The current study demonstrated that NNK-induced cytotoxicity was more pronounced in B-2A13 cells compared to BEAS-2B and B-Vector cells, and NNK dose-dependently increased γH2AX expression, the frequency of micronuclei, the S/G2-phase cell population, while no differences were observed in BEAS-2B and B-Vector cells between the treatment and control groups. This indicated that CYP2A13 played an indispensable mediating role in NNK-induced cytotoxicity and genotoxicity. Similar results were also found in B-2A13 cells treated with nicotine-free cigarette smoke extract and total cigarette smoke extract [6]. Previous studies demonstrated that NNK exposure to A549 and BEAS-2B cells did not have a significant dose/time-dependent effect on γH2AX at various test concentrations and exposure times [26,27]. This is because BEAS-2B and A549 cells show very little CYP2A6 and CYP2A13 activity [16], which are key enzymes in the metabolic activation of NNK [7].

Moreover, we found that nicotine dose-dependently inhibited cytotoxicity, DNA damage, chromosome damage, and cell cycle arrest induced by NNK in B-2A13 cells. This is in agreement with previous studies in which NNK-induced DNA damage was inhibited by nicotine in hepatic-derived cell lines, possibly due to nicotine inhibiting CYP enzymes [13]. Previous researches had reported that nicotine or nicotine metabolites inhibit various CYPs involved in the bioactivation of NNK, including CYP2A13, CYP2A6, and CYP2E1 [21]. Nicotine has a competitive inhibitory effect on CYP2A13-mediated NNK metabolism with a higher affinity [21,22]. β-nicotyrine, a nicotine-related alkaloid, has also been shown to inhibit CYP2A13 and CYP2A6 in vitro [28,29]. CYP2E1 activates nitrosamines and is competitively suppressed by nicotine in the livers of both humans and rats [30]. A molecular dynamics simulation-based study showed that nicotine had a stronger affinity for CYP2A13 than NNK [31]. Therefore, CYP2A13-catalyzed NNK metabolism may be competitively inhibited by nicotine, resulting in a decrease in NNK-induced cytotoxicity and genotoxicity. Moreover, in vitro work using purified enzymes demonstrated that CYP2A6 and CYP2A13 inactivated in the process of nicotine metabolism [12]. Further investigation found that the nicotine Δ^5′(1′)^ iminium ion is an inactivator of both CYP2A6 and CYP2A13 [32]. Hence another hypothesis is that nicotine metabolites inactivate CYP2A13 leading to a reduction in NNK-induced toxicity.

**Figure 6:**
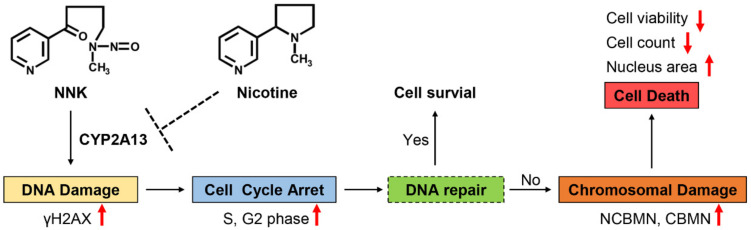
The putative schematic representation of the molecular mechanism involved in the effects of nicotine on NNK-induced toxicity in B-2A13 cells. The up and down arrows represent rising and falling respectively.

Based on the results from the study, a putative schematic representation of the molecular mechanism involving the effects of nicotine on NNK-induced toxicity in B-2A13 cells was suggested (Figure 6). This speculates that NNK is metabolized by CYP2A13 to produce toxic substances, leading to DNA damage in cells. DNA damage can activate DNA damage checkpoints, which can slow down or arrest cell cycle progression, so that cells can repair or prevent the spread of damaged chromosomes [33,34]. If the DNA damage can be repaired, the cells return to a normal cell cycle and survive, or otherwise they will lead to further chromosomal damage [35], and eventually lead to cell death [36]. Nicotine can inhibit these toxic effects of NNK by inhibiting CYP2A13.

To our knowledge, this study is the first to investigate the inhibitory effects of nicotine on the cytotoxicity and genotoxicity of NNK based on normal lung cells stably expressing CYP2A13. This study clarified the protective effect of nicotine on the toxic effect of NNK. NNK is the most well-known carcinogen in cigarette smoke, its key metabolic enzyme CYP2A13 can also metabolize many other carcinogens and/or harmful components in cigarette smoke, such as benzo-pyrene, naphthalene, and 3-methylindole, polycyclic aromatic hydrocarbons (pyrene, 1-hydroxypyrene, 1-nitropyrene and 1-acetylpyrene), and heterocyclic amines [37]. They are also thought to be associated with lung cancer [38]. Therefore, nicotine has a potential protective mechanism against carcinogenesis of various cigarette smoke components. One limitation of this study is that the molecular mechanism whereby nicotine inhibits NNK toxicity has not been fully elucidated. Therefore, future studies should consider the mechanism by which nicotine inhibits NNK-induced toxicity via enzyme activity and metabolic markers with nicotine present or absent.

## 3. Materials and Methods

### 3.1. Chemicals

NNK (>98% purity) was acquired from Toronto Research Chemicals (Toronto, ON, Canada). Nicotine (>99% purity) was obtained from the Key Laboratory of Tobacco Biological Effects (Zhengzhou, China).

### 3.2. Cell Culture and Treatment

BEAS-2B cells were acquired from the Key Laboratory of Tobacco Biological Effects (Zhengzhou, China), and cultured in Bronchial Epithelial Cell Growth Medium (BEGM, Lonza, Walkersville, MD, USA) in a humidified incubator with 5% CO_2_ at 37 °C. NNK was dissolved in dimethyl sulfoxide (DMSO) before it was used. The final exposure concentrations of NNK and nicotine were obtained by serial dilutions with the culture medium, and cells were exposed to NNK or/and nicotine in multi-well plates in a CO_2_ incubator. The final concentration of DMSO was less than 0.3%.

### 3.3. Establishment of BEAS-2B Cells Expressing CYP2A13

CYP2A13 overexpression lentivirus and empty lentivirus were purchased from GeneChem (Shanghai, China). According to the manufacturer’s instructions, BEAS-2B cells (1 × 10^5^) were cultured in six-well plates, and when the cell density reached 20%, BEAS-2B cells were infected with lentivirus. The multiplicity of infection (MOI) value was 50. The medium in the plates was discarded and complete culture medium containing lentiviral stock solution and HitransG A (Genechem, Shanghai, China) was added into plates. The medium was replaced after 16 h and the cells were observed under fluorescence microscopy to evaluate the efficiency of the infection after 72 h. Empty lentivirus and non-infected cells were used as negative controls. About 1 μg/mL of puromycin was added to each well for screening the robust cells expressing CYP2A13.

### 3.4. qRT-PCR Analysis

qRT-PCR was performed as previously described [39]. Briefly, total RNA was extracted from cells using Trizol reagent (Pufei, Shanghai, China). The RNA was reverse-transcribed into cDNA using M-MLV reverse transcriptase (Promega, Madison, WI, USA). qRT-PCR was performed using SYBR Premix Ex Taq (Takara, Shiga, Japan). The primers used were: for CYP2A13, 5′-CGCTACGGTTTCCTGCTGCTC-3′(forward) and 5′- CATCTTGGCCCGGTCCTCAA -3′ (reverse); and for beta-actin (ACTB), 5′- GCGTGACATTAAGGAGAAGC -3′ (forward) and 5′- CCACGTCACACT TCATGATGG -3′ (reverse). CYP2A13 mRNA expression was normalized to ACTB mRNA expression.

### 3.5. Western Blot Analysis

WB analysis was performed according to previously described procedures [40]. The primary antibodies against CYP2A13 (Invitrogen, PA5-101310) and GAPDH (Cell Signaling Technology, 97166S) were diluted at a ratio of 1:1000. The secondary antibodies, Goat anti-rabbit IgG-HRP (Cell Signaling Technology, 7074S,) and Horse anti-mouse IgG-HRP (Cell Signaling Technology, 7076S), were diluted at a ratio of 1:3000. The reference protein GAPDH was used to calibrate the errors generated by sample loading.

### 3.6. Assessment of Cytotoxicity

CCK-8 assay: Cell cytotoxicity was determined using the Cell Counting Kit-8 (CCK-8, Dojindo, Kumamoto, Japan) [41]. Approximately 1 × 10^4^ cells were plated into each well of a 96-well microplate. After 24 h of incubation in a CO_2_ incubator at 37 °C, the original medium was aspirated and a fresh medium containing NNK and/or nicotine was added. After 24 h of treatment, 10% (*v*/*v*) CCK-8 solution was added and incubated at 37 °C for 3 h. The optical density (OD value) was measured at a wavelength of 450 nm with a microplate reader (Molecular Devices LLC, San Jose, CA, USA).

HCS: The experimental protocol for HCS was previously described [42,43]. Approximately 1 × 10^4^ cells were plated into each well of a PhenoPlate 96-well microplate (PerkinElmer, Waltham, MA, USA). After 24 h of incubation in a CO_2_ incubator at 37 °C, the original medium was aspirated and a fresh medium containing a combination of NNK and nicotine was added. After 24 h of treatment, the B-2A13 cells were stained with Hoechst 33342 (Beyotime, Shanghai, China). Hoechst 33342 can measure features such as cell count and nuclear area by labeling DNA. Cell imaging with fluorescence analysis was performed on a PerkinElmer Operetta CLS High Content Screening platform with Harmony 4.5 software (PerkinElmer, Waltham, MA, USA).

### 3.7. γH2AX Assay

The γH2AX assay was modified on the basis of the previous experimental method [44]. Approximately 1 × 10^4^ cells were plated into each well of a PhenoPlate 96-well microplate (PerkinElmer, Waltham, MA, USA), and 24 h later cells were continuously exposed to NNK or a combination of NNK and nicotine for 24 h. The cells were then washed with phosphate-buffered saline (PBS) fixed with 4% paraformaldehyde (Beyotime, Shanghai, China), permeabilized with immunostaining permeable fluid (Beyotime, Shanghai, China), and blocked with 5% bovine serum albumin (BSA, Sigma-Aldrich, St. Louis, MO, USA). After blocking, the cells were incubated with 100 μL of 0.1% (*v*/*v*) Phospho-Histone H2A.X (Ser139) Antibody (Cell Signaling Technology, Danvers, MA, USA) in 1% BSA overnight at 4 °C. After being washed three times with PBS, the cells were incubated with 100 μL of 0.1% (*v*/*v*) Anti-rabbit IgG (H + L), F(ab’)2 Fragment (Alexa Fluor^®^ 647 Conjugate) (Cell Signaling Technology, Danvers, MA, USA) in 1% BSA for 2 h at room temperature in darkness. Next, 100 μL of Hoechst 33342 (Beyotime, Shanghai, China) was added to each well for 10 min. After the wells were washed with PBS, 100 μL of PBS was added per well and the plates were immediately placed on the PerkinElmer Operetta CLS High Content Screening platform for analysis.

### 3.8. Micronucleus Assay

The experimental protocol was modified according to the method previously described [43,45,46]. CBMN assay: Approximately 1 × 10^4^ cells were plated into each well of a PhenoPlate 96-well microplate (PerkinElmer, Waltham, MA, USA). After 24 h of incubation in a CO_2_ incubator at 37 °C, the original medium was aspirated and a fresh medium containing NNK or a combination of NNK and nicotine with cytochalasin B (4 μg/mL) was added. After 24 h of treatment, cells were washed with PBS, fixed with 4% paraformaldehyde (Beyotime, Shanghai, China), and stained with Hoechst 33342 (Beyotime, Shanghai, China) and Cell Mask Red (Life Technology, Carlsbad, CA, USA).

NCBMN assay: Approximately 1 × 10^4^ cells were plated into each well of a PhenoPlate 96-well microplate (PerkinElmer, Waltham, MA, USA). After 24 h of incubation in a CO_2_ incubator at 37 °C, the original medium was aspirated and a fresh medium containing NNK or a combination of NNK and nicotine was added for 24 h. The cells were then washed with PBS and stained with Hoechst 33342 for 10 min in the dark.

After being washed with PBS, 100 μL of PBS was added to each well and the plates were immediately placed on the PerkinElmer Operetta CLS High Content Screening platform for analysis. Frequencies of micronuclear cells were measured in at least 2000 binuclear cells (CBMN assay) or at least 2000 cells (NCBMN assay) per sample.

### 3.9. Cell Cycle Analysis

The cell cycle distribution was measured using the cell cycle and apoptosis analysis kit (Beyotime, Shanghai, China) [47]. Approximately 3 × 10^5^ cells were seeded into each well of a 6-well culture plate, and 24 h later the cells were continuously exposed to NNK or a combination of NNK and nicotine for 24 h. Cells were harvested by trypsinization, centrifuged, and the pellets were resuspended in 1 mL of cold PBS. They were then centrifuged and the pellet was fixed in 75% ice-cold ethanol at 4 °C overnight. After the fixative solution was aspirated, the cells were centrifuged and the pellet was incubated in propidium iodide (PI) and RNase for 30 min. The cell cycle was analyzed by FACS flow cytometer Accuri ^TM^ C6 Plus (BD Biosciences, San Jose, CA, USA), and the results were processed using the FlowJo (v10.8.1, BD Bioscience, San Jose, CA, USA) software program.

### 3.10. Statistical Analysis

GraphPad Prism 8.0 software (GraphPad Software, San Diego, CA, USA) was used for data analysis, the results were presented as mean ± standard deviation (SD). A two-tailed Student’s *t*-test was used to determine significant differences (*p* < 0.05). All experiments were repeated at least three times.

## 4. Conclusions

To elucidate the effect of nicotine on the cytotoxicity and genotoxicity of NNK in lung cells, BEAS-2B cells stably expressing CYP2A13 were constructed and a variety of in vitro toxicological indicators were tested in this study. The results showed that CYP2A13 played an indispensable mediating role in cytotoxicity and genotoxicity of NNK. Nicotine inhibited cytotoxicity and multi-dimensional genotoxicity in NNK-induced B-2A13 cells, possibly by inhibiting CYP2A13. This study provided mechanistic insights into the protective effects of nicotine, and suggested that nicotine has a potential protective mechanism against carcinogenesis of various cigarette smoke components metabolized by CYP2A13. In addition, it suggested that nicotine may influence toxic effects of some non-carcinogenic harmful ingredients activated by CYP2A13 and CYP2A6 enzymes, and then affect the health risks of tobacco products.

## Data Availability

The data presented in this study are available on request from the corresponding author.

## Sample Availability

Samples of the compounds are not available from the authors.
